# Effects of Litter Size and Parity on Farrowing Duration of Landrace × Yorkshire Sows

**DOI:** 10.3390/ani12010094

**Published:** 2021-12-31

**Authors:** Mingming Ju, Xiaonv Wang, Xinjian Li, Menghao Zhang, Lidan Shi, Panyang Hu, Ben Zhang, Xuelei Han, Kejun Wang, Xiuling Li, Lisheng Zhou, Ruimin Qiao

**Affiliations:** 1 College of Animal Science and Technology, Henan Agricultural University, Zhengzhou 450002, China; jmm626@163.com (M.J.); xiaonv1017@163.com (X.W.); lxjlongfei@163.com (X.L.); zhangmenghao2021@163.com (M.Z.); sld1014@163.com (L.S.); hpy9809@163.com (P.H.); zbandfs@163.com (B.Z.); hxl014@126.com (X.H.); wangkejun.me@163.com (K.W.); xiulingli@henau.edu.cn (X.L.); 2 College of Animal Science, Qingdao Agricultural University, Qingdao 266109, China; zls123668@qau.edu.cn

**Keywords:** duration of farrowing, parity, litter size, Landrace × Yorkshire sows

## Abstract

**Simple Summary:**

Litter size is an important economic trait in pigs. Improving the number born alive is an important breeding goal of the pig husbandry. A shorter farrowing duration is welcome for facilitating the management and sows’ health. Therefore, the aim of this study was to explore the effect of litter size and parity on farrowing duration, to determine whether a shorter length of farrowing duration could be considered as a breeding parameter in pig breeding. Our results showed the total number born had no significant relation with farrowing duration, but number of stillbirths increased with the prolongation of farrowing duration and decrease of live litter size if farrowing duration was longer than 240–300 min. Different parities sows had little difference in the same farrowing duration interval except for gilts. A shorter farrowing duration within 300 min might be considered in pig breeding without worrying about the decreasing of live litter size or the negative effect of parity.

**Abstract:**

Litter size has increased and farrowing duration has also prolonged in recent years. The aim of this study was to analyze the effect of litter size and parity on farrowing duration (FAR) to estimate the possibility of selecting a short farrowing duration. We recorded 32,200 parturitions of 8420 Landrace × Yorkshire sows, determined farrowing duration, litter size, parity, gestation length. Results showed that total number of born (TNB) and parity obeyed a cubic (*p* = 0.0004, *p* = 0.004) relationship while number born alive (NBA) and number born dead (NBD) obeyed a linear (*p* = 0.0239, *p* = 0.0035) relationship with FAR. Gestation length obeyed a linear (*p* = 0.02) relationship with FAR. FAR of sows with stillbirth was longer than that of sows without stillbirth. Stillbirth rate increased rapidly from about 2% to 4%, especially when FAR was over 240 min. FAR gradually prolonged with the parities. FAR of 7th parity sows was longer than that of 1st~6th parity sows (*p* < 0.05), but different parity sows had little difference in the same FAR interval except for gilts. Results indicated it was possible and necessary to consider FAR into pig breeding without worrying about decreasing of live litter size or negative effect of parity if FAR was shorter than 300 min.

## 1. Introduction

Reproductive traits are the most important concepts in determining the output of a pig farms. Litter size is an important reproductive trait, and it is one of the decisive factors affecting the economic benefits of the entire pig industry. Over tens of reproduction traits are considered in calculating the breeding index to rank and select breed pigs. However, the average stillbirth rate of piglets was still 3–8%, of which approximately 75% happened during parturition [1], the increase of stillbirth rate would inevitably reduce the profits of pork production [2,3]. Larger litter size may lead to a longer farrowing duration (FAR) and is related to the lower birth weight of piglets [4]. It has been suggested that large litter size can also lead to the increase of stillbirth rate, which is likely to bring significant negative impact on animal welfare [5]. Farrowing is known to be a stressful process of both piglets and sows, and a longer duration of farrowing process often requires extra care from nursing staff and sometimes impairs the uterus health of sows, which would bring certain difficulties in management and even a fertility reduction of sows during the next parturition. Many stillbirths occur during parturition due to dystocia and prolong farrowing.

Duration of farrowing (FAR) was reported to prolong with the number of stillbirths [6]. A prolonged FAR is said to impair fertility in pigs [7], as sows with a longer FAR tended to have a higher risk of post parturient disorders [8]. However, at the same time, the prolongation of FAR was also in relation to the increase of litter size [9,10]. In addition, genetic, breed, age, length of gestation, feeding management, environment and nutritional factors could also affect FAR [11]. There have been some studies focused on the environment conditions control in the farrowing house in order to ensure the smooth production of piglets [12,13,14].

The aim of this study was to explore the exact effect of litter size including total number born, number born alive and number dead on FAR. We also wanted to evaluate the impact of parity on FAR and litter size in order to evaluate the possibility of short FAR as a breeding indicator for pigs.

## 2. Material and Methods

### 2.1. Animals

The observational study was conducted in 2017–2018 on a commercial pig farm in Central China. Parturitions (*n* = 32,231) of 8420 randomly selected healthy Landrace × Yorkshire hybrid sows between 1st and 10th parity were included in this study. The sows farrowed in 26 batches. Pregnant sows were fed 1.8–2.0 kg/day for the first 7 days; 2.0–2.5 kg/day from days 7 to 30; 2.5–3.0 kg/day from days 30 to 70; 2.5 kg/day from days 70 to 90; and 3.5 kg/day from days 90 to 110. The diets were formulated according to their body condition. During 3 days before farrowing, sows were fed 2.5 kg/day. On the day of farrowing, sows were fed 0.5–1.5 kg/day. For the first 5 days after parturition, sows were fed 2.0, 2.5, 3.0, 3.5, and 4.0 kg/day, respectively.

All pregnant sows were fed in groups and transferred to farrowing houses 1 week before farrowing and fed in single crates in farrowing houses with free access to drinking water. The farrowing pens were 2.50 × 2.30 m^2^ in size, the farrowing crates were 2.10 × 0.70 m^2^ in size. The average room temperature was 25 °C. The environment of this pig farm was controlled by Big Dutchman ventilation system and cooling pad in summer and warm air heating system in winter. All sows were raised under regular disinfection and vaccination procedures (Table 1).

### 2.2. Measurements

Duration of farrowing (FAR) was defined as the time interval between the first birth to the complete expulsion of placenta. We also determined the following sow traits: farrowing batch, farrowing barn, farrowing house, farrowing pen, the length of pregnancy, parity, feeders and nursing staffs. The length of pregnancy was defined as the time interval between fertilization and farrowing. Total number born piglets (TNB), number of born alive piglets (NBA) and number of born dead piglets (NBD) in each litter were investigated by nursing staffs. TNB consisted of NBA, NBD and mummies. Piglets that had no breathing and heartbeat at birth, or had heartbeat but no breathing and unable to breathe after rescue, were defined as NBD.

FAR and delivery mode (normal delivery and assisted delivery) were recorded by the nursing staff who works day and night shifts in farrowing houses. Nursing staffs’ job is for delivery and nursing. When the sow’s amniotic fluid is out, they need to wipe the sow’s udder and hind body with warm water and disinfect with 0.1% potassium permanganate solution and wait for the sow to farrow. They need to keep an eye on each sow in farrowing and keep an eye on each sow as she gives birth, in order to deal with the dystocia of sows, the suspended death of newborn piglets, and to cut umbilical cord, to assist the piglets to keep warm and access the colostrum as soon as possible.

The assisted delivery was adopted if sows had strong parturition symptoms such as giving an expulsion effort but no piglet was delivered after 1–2 h of amniotic fluid outflow, or a fetus was present in canal or uterine cervix but sows had not enough labor force or the litter interval was more than 1 h. The assisted delivery consists of an injection of 0.5–1.0 mL oxytocin or and an artificial assisted delivery.

### 2.3. Statistical Analysis

All the data was analyzed by SPSS Statistics 26 (IBM SPSS Statistics, IBM Corp, Armonk, NY, USA) and presented with R software. Variables including FAR, TNB, NBA, NBD, parity (P) and gestation length (G) were checked for normal distribution. An initial univariable screening was performed to identify potential influencing factors of FAR and litter size. The correlations of FAR and TNB, NBA, NBD, P and G were tested and estimated. The regression model that had the highest fitting degree and achieved significance level was selected. Based on the significance levels of those tests, a multivariable linear model for FAR was created. Farrowing house, farrowing pen, nursing staff, feeder, and batch were included as random factors and delivery mode as fixed effects. The correlations of parity (P) and gestation length (G) on litter size were tested and estimated. Based on the significance levels of those tests, a multivariable linear model for TNB and NBA and a lineal model for NBD were created. Farrowing house, farrowing pen, nursing staff, feeder, and batch were included as random factors and delivery mode as a fixed effect. Results were presented as Mean ± SD and considered significant at *p* ≤ 0.05.

## 3. Results

Parturition records of FAR longer than 10 h (*n* = 225), live litter size less than 5 (*n* = 1172) and more than 19 (*n* = 31) were excluded. Data of parity ≥ 7 were classified as parity 7. A total of 32,200 parturitions of 8420 Landrace × Yorkshire hybrid sows were included in the following analysis: 3451 assistant delivery parturitions (10.7%). Distribution of farrowing duration, litter size, parity and gestation length were presented in Figure S1.

### 3.1. Effects of Litter Size on Farrowing Duration

**Figure 1:**
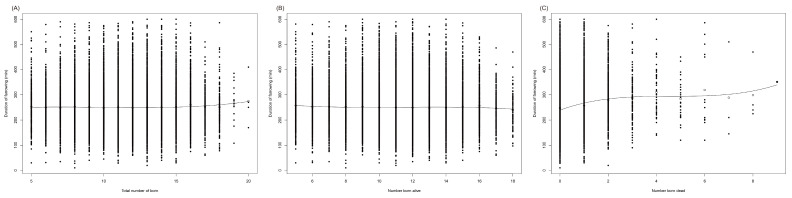
Relation farrowing duration to litter size. Cubic relation between total number of born (**A**), number born alive (**B**), number born dead (**C**) and farrowing duration.

Total number of born (TNB) (5–20), number born alive (NBA) (5~18) and number born dead (NBD) (0–9) obeyed a cubic (R^2^ = 0.77, *p* = 0.0004; R^2^ = 0.54, *p* = 0.0442; R^2^ = 0.79, *p* = 0.0043) relationship with farrowing duration (FAR) (10.2–600 min) (Figure 1). In the final multivariable model for FAR, the interact of TNB and NBA (TNB × NBA) were significant (*p* = 0.012). The average FAR was 250.05 ± 67.63 min, TNB was 11.61 ± 2.45, NBA was 11.29 ± 2.42, and NBD was 0.26 ± 0.58, respectively. An overview of effect of litter size on FAR was listed in Figure 1 and Table 2, Table 3 and Table 4.

From Table 2, farrowing duration indicated no significant difference between different TNB, but significant difference between different NBA (*p* < 0.05). Sows of different TNB and NBA had almost the same FAR variations, about 27%. The length of FAR first decreased and then increased with the increase of NBA. When litter size was less than 11, NBA took a little longer FAR than TNB. When litter size was over 12, NBA generally took a shorter time to farrow than TNB. Sows with 5 and 16 NBA had the longest FAR of 257.49 ± 76.8 min and 256.62 ± 67.61 min, longer than sows with 13, 17 and 18 NBA. Sows with 18 NBA had the shortest FAR of 239.98 ± 65.88 min, which was shorter than that of all the other sows, followed by sows with 17 and 13 NBA of 246.21 ± 65.05 min and 247.19 ± 65.89 min.

From Table 3, 78.69% parturitions (*n* = 25,339) had no NBD. 17.95% parturitions (*n* = 5779) had 1 NBD. 2.56% parturitions (*n* = 824) had 2 NBD. 0.50% parturitions (*n* = 162) had 3 NBD. 0.14 % parturitions (*n* = 44) had 4 NBD. 0.09% parturitions (*n* = 30) had 5 NBD. 0.04% parturitions (*n* = 14) had 6 NBD. 0.01% parturitions (*n* = 3) had 7 NBD. 0.01% parturitions (*n* = 4) had 8 NBD. 1 parturition had 9 NBD. Farrowing duration indicated significant difference between sows with different NBD (*p* < 0.05). Length of FAR of sows with 0, 1 and 5 NBD had about 27% variability. Length of FAR of sows with 6, 7 and 8 NBD had over 38% variability. FAR prolonged with NBD from 0 to 4. FAR of sows with 0 NBD were 62.64 min longer than that of sows with 4 NBD (247.18 ± 66.02 min vs. 309.82 ± 99.09 min) (*p* < 0.05). Sows with 6 NBD had the longest FAR, 72 min and 63 min longer than sows with 0 NBD and 1 NBD (319.24 ± 153.66 min vs. 247.18 ± 66.02 min, 256.60 ± 68.44 min) (*p* < 0.05).

From Table 4, after dividing FAR into 5 groups: 0–180 min, >180–240 min, >240–300 min, >300–360 min and >360–600 min, number born dead and stillbirths rate increased with the length of FAR, the longer the FAR, the more the NBD (from 0.21 to 0.45) and stillbirth rate (from 1.83% to 0.45%), especially when FAR was longer than 240 min. When the length of FAR was over 240 min, there would be significantly more NBD (*p* < 0.05).

### 3.2. Effects of Parity on Farrowing Duration and Litter Size

**Figure 2:**
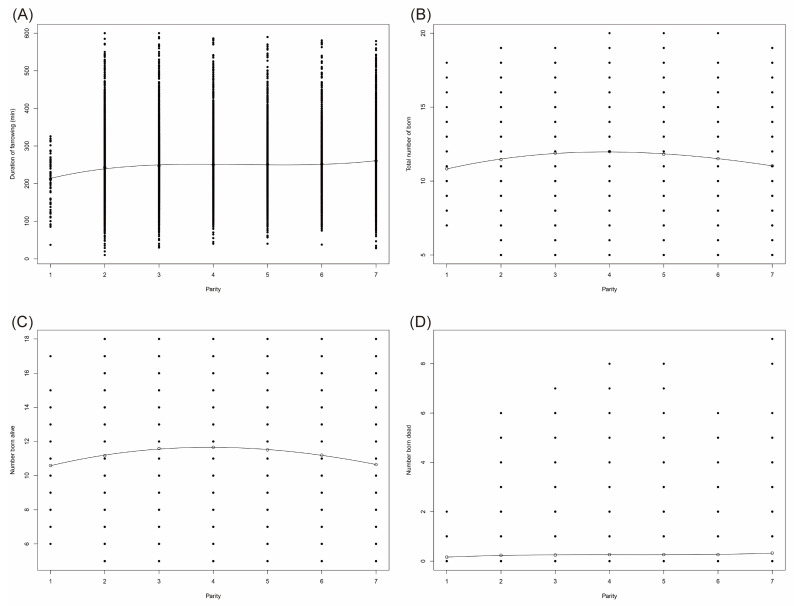
Cubic relation between parity and farrowing duration and litter size. Cubic relation between parity and farrowing duration (**A**), total number born (**B**), number born alive (**C**) and number born dead (**D**).

Parity obeyed a cubic relationship with FAR (R^2^ = 0.98, *p* = 0.0043), TNB (R^2^ = 0.997, *p* = 0.0003), NBA (R^2^ = 0.997, *p* = 0.0002) and NBD (R^2^ = 0.979, *p* = 0.0052) (Figure 2). An overview was shown in Figure 2 and Table 5 and Table 6. From Table 5, FAR gradually prolonged with parity, from 212.65 ± 61.55 min to 260.42 ± 68.02 min. 1st parity sows (*n* = 89) had a FAR of 212.65 ± 61.90 min, 2nd parity sows (*n* = 6915) of 243.01 ± 67.68 min, 3rd parity sows (*n* = 7477) of 247.23 ± 67.23, 4th parity sows (*n* = 5078) of 250.23 ± 65.84, 5th parity sows (*n* = 3559) of 251.10 ± 67.28 min, 6th parity sows (*n* = 3065) of 252.01 ± 68.75 min, 7th parity sows (*n* = 6018) of 260.40 ± 68.02 min, respectively. Sows of parity 1 and 2 had a significantly shorter FAR, while sows of parity 6 and 7 had a significantly longer FAR (*p* < 0.05).

TNB and NBA increased from 10.83 ± 2.35 and 10.59 ± 2.34 to 11.99 ± 2.44 and 11.66 ± 2.39 with the increase of the first four parities, and decreased to 11.03 ± 2.38 and 10.65 ± 2.34 with the increase of the last four parities. NBD always increased as the increase of parities, from 0.16 ± 0.40 to 0.32 ± 0.65, by 0.16 ± 0.25. Sows of parity 4 had the most TNB (11.99 ± 2.44) and NBA (11.66 ± 2.39). There was no significant difference among TNB and NBA of sows in parity 3 to 5, or among NBD of sows in parity 2 to 6. Sows of parity 3, 4 and 5 had significant more TNB and NBA than sows of parity 1, 2, 6 and 7 (*p* < 0.05). Sows of parity 7 had significant more NBD than sows of parity 1, 2 and 3 (*p* < 0.05).

Then, we looked into if there was significant variation among seven parities within each of the 5 FAR groups: 0–180 min, >180–240 min, >240–300 min, >300–360 min and >360–600 min (Table 6). Significant differences were detected when FAR was of 0–180 min, >240–300 min, and >300–360 min. When sows had FAR of 0~180 min and of >300–360 min, gilts had a shorter FAR than sows of all the other 6 parities (*p* < 0.05). When sows had FAR of >240–300 min, gilts had a shorter FAR than sows of parities 2 to 6 (*p* < 0.05).

### 3.3. Effect Gestation Length on Farrowing Duration and Litter Size

**Figure 3:**
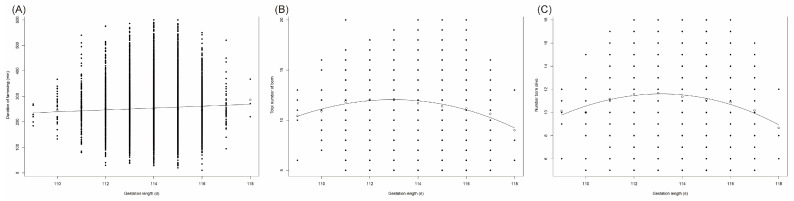
Relation between gestation length and farrowing duration and litter size. Relation between farrowing duration (**A**), total number born (**B**) and number born alive (**C**) with gestation length.

Gestation length (109–118 days) obeyed a lineal (R^2^ = 0.51) relationship with FAR, TNB (R^2^ = 0.946, *p* = 0.0004) and NBA (R^2^ = 0.915, *p* = 0002) (Figure 3). No significant relationship was found between gestation length and NBD. The average gestation length (G) was 114.28 ± 0.98 days. FAR was positively associated with length of gestation (β = 3.67; *p* = 0.02). An overview was shown in Table 7. There were only seven and three parturitions of gestation length of 109 days and 118 days. Additionally, significant difference was only found among sows of gestation length of 109 days and 118 days for FAR, TNB and NBA. After removing these 10 parturitions records, there was no difference in FAR between 110–117 days of gestation length. TNB and NBA of sows with 112 and 113 days of gestation were significantly higher than those in other gestations.

## 4. Discussion

A longer duration of farrowing (FAR) is thought to be against the health of sows [15] and their next delivery performance which would reduce the working efficiency for feeders and nursing staffs in farrowing houses. Some studies also suggested a positive correlation between FAR and litter size, the longer FAR, the more litter size. Therefore, in this study, we investigated the influence of litter size including TNB, NBA and NBD on FAR and the impact of parity on both FAR and litter size using a large scale of farrowing data in order to look into whether it was reasonable to consider a shorter FAR as one of the breeding parameters in pigs in the further.

By analyzing 32,200 parturition records from 8420 Landrace × Yorkshire sows, we found that the average length of FAR was about 250 min, or about 4.2 h, which was another piece of evidence that the FAR is getting longer and longer. In 2004, FAR was reported to last 133 min [9], and increased to 166 min [6] and 268 min [12], though the common use of oxytocin-like compound in pig production can shorten the length of FAR [16]. This might be partly because of the rapid increase of litter size in pigs since 2004. The average length of FAR in this study was significantly shorter than the 6~8 h of a batch of European superior sows [17]. Average TNB in this study was 11 compared to as high as 20 in the most hyper prolific sows in Europe.

In this study, we found that the litter size and parity had an impact on FAR by using the multivariable linear model. The interaction between TNB and NBA were detected. In the study of Bjorkman et al., they used the multinomial logistic regression model to explore the relationship between litter size, parity and farrowing duration. They found a positive correlation between number born alive and FAR, and number of stillbirth was significantly correlated with FAR [18]. The coefficient of variation of TNB and NBA were larger, about 27%. Significant difference was not found in FAR between different TNB, but found between different NBA because of the interact between FAR and stillbirth [19]. Shortening the length of FAR will not reduce the effective NBA. FAR of >240 to 300 min might be the ideal cut-off point in this population (with 5 to 22 litter size). This was in coincidence with the general believing that FAR that exceeded 300 min was likely to cause dystocia and new-born piglet complications, and piglets that experienced a longer FAR are more likely to die at birth [17,20].

Besides, NBD increased gradually with FAR, especially when FAR was longer than 240 min, the NBD increased rapidly and linearly. Studies have shown that a longer FAR could affect perinatal mortality or subsequent piglet growth. FAR and the birth order of piglets were reported to be the main factors determining the risk of stillbirth [20]. Some studies also pointed that there was a positive correlation between the number of born alive and FAR, and the number of stillbirths was significantly negatively correlated with FAR [18,21].

In addition, we found that parity had a significant impact on FAR. As the parity increased, FAR gradually became longer. Studies show that parity has a slight effect on the number of colostrum metabolites, which might affect the reproduction performance of sows, including litter weight at birth and piglet mortality [22]. Parity and FAR could affect the incidence of postpartum disease in sows; if FAR was longer than 4 h, the sows would be at greater risk for fever 1 day after parturition [23], which affected the reproductive performance of sows and sped up the sows’ elimination. Besides, the effect of parity on FAR might be due to the aging of sows’ uteri after multiple parturitions, which weakens their muscles’ ability to contract during parturitions, resulting in prolonged FAR.

Some studies have shown that FAR was not only affected by parity, but also by the sow’s physical condition, feeding and environment. For example, a higher energy intake in sows during late gestation could improve the farrowing duration traits [24], adding the *Bacillus* during the perinatal period of sows could shorten FAR and the weaning-estrous interval [25]. FAR was positively correlated with the gestation, and FAR with 118 day of pregnancy was the longest. However, there was no significant difference between gestation (110–117 days) and FAR after removing the data of pregnancy was 109 and 118. A study reported that the length of gestation (112–119 days) was negatively correlated with FAR, but the effect of litter size on FAR was not considered [6].

In general, we found that litter size had an impact on FAR, and there was a positive correlation between parity and FAR. The total number born and the number born alive increased gradually in 1–4th parity. All of these indicated that we can select sows with a shorter FAR and of the 1–4th parity during pig breeding.

## 5. Conclusions

In summary, we found that number of stillbirths increased with the increase of farrowing duration and decrease live litter size if farrowing duration was longer than 240–300 min. It might be possible to choose a shorter farrowing duration without compromising decreasing litter size in pig breeding. In order to further confirm the effect of litter size and parity on farrowing duration, it is necessary to further explore it from the perspective of genome.

## Figures and Tables

**Table 1:** Immunization procedure of Landrace × Yorkshire sows used in this study.

Stage	Age (Day)	Vaccine
Piglets	1	Swine Pseudorabies Vaccine (Intranasal drop)
	7	Swine Mycoplasma Hyopneumoniae Vaccine
	10	Swine Atrophic Rhinitis Vaccine
	12	Highly Pathogenic Porcine Reproductive and Respiratory Syndrome Vaccine
	22	Classical Swine Fever Vaccine
	52	Swine Pseudorabies Vaccine
	59	Swine Foot and Mouth Disease (Type O) vaccine
	66	Classical Swine Fever Vaccine
Gilts	150	Porcine Parvovirus Disease Vaccine and Swine Epidemic Encephalitis Vaccine
	157	Classical Swine Fever Vaccine
	164	Swine Pseudorabies Vaccine
	171	Swine Foot and Mouth Disease (Type O) vaccine
	178	Porcine Cirovirus Type 2 Vaccine
	185	Porcine Parvovirus Disease Vaccine and Swine Epidemic Encephalitis Vaccine
	192	Highly Pathogenic Porcine Reproductive and Respiratory Syndrome Vaccine
	199	Swine Foot and Mouth Disease (Type O) vaccine
	206	Classical Swine Fever Vaccine
	213	Swine Pseudorabies Vaccine

**Table 2:** Descriptive statistics of farrowing duration of different total number of born and number born alive piglets.

Litter Size	Number of Litters	Farrowing Duration (min)	CV (%) ^1^
	Total Number of Born/Number Born Alive		
5	418/573	252.80 ± 72.75/257.49 ^a^ ± 76.8	28.78/29.83
6	673/767	249.43 ± 69.29/251.90 ^ab^ ± 72.61	27.78/28.83
7	889/1085	248.34 ± 67.93/251.96 ^ab^ ± 69.26	27.35/27.49
8	1685/1823	252.74 ± 69.33/253.41 ^ab^ ± 69.17	27.43/27.29
9	1809/2283	251.64 ± 69.86/253.47 ^ab^ ± 69.29	27.76/27.34
10	4314/4586	247.93 ± 66.46/249.54 ^ab^ ± 67.94	26.81/27.23
11	4443/4737	250.63 ± 67.27/249.68 ^ab^ ± 67.38	26.84/26.99
12	6469/6751	247.90 ± 65.86/248.89 ^ab^ ± 66.03	26.57/26.53
13	4643/4077	249.81 ± 68.08/247.19 ^bc^ ± 65.89	27.25/26.65
14	3494/2983	250.32 ± 66.76/248.83 ^ab^ ± 67.15	26.67/26.99
15	1882/1524	251.81 ± 68.67/252.00 ^ab^ ± 68.02	27.27/26.99
16	960/676	260.09 ± 70.51/256.62 ^a^ ± 67.61	27.11/26.34
17	326/239	251.65 ± 68.57/246.21 ^bc^ ± 65.05	27.25/26.42
18	177/96	250.82 ± 77.24/239.98 ^c^ ± 65.88	30.79/27.45
19	14/-	270.17 ± 77.51/-	28.69/-
20	4/-	274.95 ± 99.80/-	36.30/-

^1^ CV represents coefficient of variation of duration of farrowing. ^a–c^ Values within a column with different superscripts differ significantly at *p* < 0.05.

**Table 3:** Descriptive statistics of farrowing duration of different number born dead piglets.

Number of Stillborn Piglets	Number of Litters (Frequency %)	Farrowing Duration (Minute)	CV (%) ^1^
0	25,339 (78.69)	247.18 ^c^ ± 66.02	26.71
1	5779 (17.95)	256.60 ^bc^ ± 68.44	26.67
2	824 (2.56)	279.01 ^abc^ ± 84.27	30.20
3	162 (0.50)	287.14 ^abc^ ± 87.34	30.42
4	44 (0.14)	309.82 ^ab^ ± 99.09	31.98
5	30 (0.09)	283.98 ^abc^ ± 79.20	27.89
6	14 (0.04)	319.24 ^a^ ± 153.66	48.13
7	3 (0.01)	288.40 ^abc^ ± 194.63	67.49
8	4 (0.01)	298.65 ^abc^ ± 114.99	38.50
9	1 (-)	251.00	

^1^ CV represents coefficient of variation of duration of farrowing. ^a–c^ Values within a column with different superscripts differ significantly at *p* < 0.05.

**Table 4:** Descriptive statistics of number born dead piglets of different farrowing duration.

Farrowing Duration (Minute)	Number of Litters (Frequency %)	Number Born Dead	Stillbirth Rates
0–180	4192 (13.02)	0.21 ^d^ ± 0.52	1.83%
>180–240	10,607 (32.94)	0.23 ^d^ ± 0.54	1.96%
>240–300	11,621 (36.09)	0.25 ^c^ ± 0.55	2.19%
>300–360	3980 (12.36)	0.33 ^b^ ± 0.65	2.85%
>360–600	1800 (5.59)	0.45 ^a^ ± 0.86	3.87%

^a–d^ Values within a column with different superscripts differ significantly at *p* < 0.05.

**Table 5:** Descriptive statistics of farrowing duration and litter size of different parities.

Parities	Number of Litters	Farrowing Duration (Minute)	CV (%) ^1^	TNB ^2^	NBA ^3^	NBD ^4^
1	89	212.65 ^d^ ± 61.90	29.12%	10.83 ^c^ ± 2.35	10.59 ^c^ ± 2.34	0.16 ^c^ ± 0.40
2	6915	243.01 ^c^ ± 67.68	27.85%	11.45 ^b^ ± 2.51	11.17 ^b^ ± 2.48	0.23 ^b^ ± 0.53
3	7477	247.23 ^bc^ ± 67.23	27.19%	11.89 ^a^ ± 2.42	11.59 ^a^ ± 2.40	0.24 ^b^ ± 0.55
4	5078	250.23 ^bc^ ± 65.84	26.31%	11.99 ^a^ ± 2.44	11.66 ^a^ ± 2.39	0.26 ^ab^ ± 0.60
5	3559	251.10 ^bc^ ± 67.23	26.79%	11.81 ^a^ ± 2.42	11.51 ^a^ ± 2.39	0.26 ^ab^ ± 0.60
6	3065	252.01 ^b^ ± 68.35	27.12%	11.52 ^b^ ± 2.34	11.20 ^b^ ± 2.33	0.26 ^ab^ ± 0.56
7	6018	260.40 ^a^ ± 68.02	26.12%	11.03 ^c^ ± 2.38	10.65 ^c^ ± 2.34	0.32 ^a^ ± 0.65

^1^ CV represents coefficient of variation of duration of farrowing. ^a–d^ Values within a column with different superscripts differ significantly at *p* < 0.05. ^2^ TNB represents total number of born. ^3^ NBA represents number born alive. ^4^ NBD represents number born dead.

**Table 6:** Descriptive statistics of different groups of farrowing duration of different parities.

Parities	Duration of Farrowing (Minutes)				
	0–180	>180–240	>240–300	>300–360	>360–600
1	129.40 ^b^ ± 34.00	215.62 ± 14.58	263.90 ^b^ ± 13.83	314.90 ^b^ ± 8.04	
2	149.21 ^a^ ± 26.81	213.02 ± 15.91	266.97 ^ab^ ± 15.91	326.13 ^a^ ± 16.10	412.68 ± 48.62
3	149.24 ^a^ ± 26.68	213.52 ± 15.84	267.84 ^a^ ± 15.79	324.58 ^a^ ± 16.41	420.94 ± 52.43
4	149.69 ^a^ ± 25.29	213.84 ± 15.90	268.00 ^a^ ± 15.73	325.84 ^a^ ± 16.60	418.98 ± 53.50
5	149.95 ^a^ ± 24.59	213.22 ± 15.84	268.25 ^a^ ± 16.20	326.44 ^a^ ± 16.14	414.80 ± 51.88
6	150.02 ^a^ ± 24.35	214.41 ± 16.02	267.67 ^a^ ± 15.94	325.98 ^a^ ± 16.49	419.70 ± 52.82
7	148.65 ^a^ ± 26.95	214.24 ± 15.99	268.92 ^a^ ± 16.08	325.78 ^a^ ± 16.66	414.50 ± 45.60

^a, b^ Values within a column with different superscripts differ significantly at *p* < 0.05.

**Table 7:** Descriptive statistics of farrowing duration and litter size of different gestation length.

Gestation Length (day)	Number of Litters	Farrowing Duration (min)	Total Number of Born	Number Born Alive
109	7	224.23 ^b^ ± 33.09	10.43 ^ab^ ± 2.23	10.14 ^ab^ ± 2.12
110	37	246.84 ^ab^ ± 59.11	10.89 ^a^ ± 2.04	10.00 ^ab^ ± 2.44
111	250	257.78 ^ab^ ± 71.92	11.86 ^a^ ± 2.39	11.17 ^a^ ± 2.40
112	1200	251.39 ^ab^ ± 73.80	12.05 ^a^ ± 2.30	11.58 ^a^ ± 2.34
113	4403	248.93 ^ab^ ± 69.98	12.08 ^a^ ± 2.29	11.67 ^a^ ± 2.28
114	11,789	250.29 ^ab^ ± 67.58	11.69 ^a^ ± 2.40	11.37 ^a^ ± 2.36
115	12,457	250.54 ^ab^ ± 66.48	11.39 ^a^ ± 2.50	11.12 ^a^ ± 2.48
116	1997	246.02 ^ab^ ± 65.05	11.16 ^a^ ± 2.62	10.89 ^a^ ± 2.59
117	57	261.78 ^ab^ ± 74.62	10.63 ^a^ ± 2.42	10.19 ^ab^ ± 2.55
118	3	286.60 ^a^ ± 74.90	9.00 ^b^ ± 3.61	8.67 ^b^ ± 3.06

^a, b^ Values within a column with different superscripts differ significantly at *p* < 0.05.

## Data Availability

All data, models, or code generated or used during the study are available from the corresponding author by request.

## Supplementary Materials

The following supporting information can be downloaded at: https://www.mdpi.com/article/10.3390/ani12010094/s1, Figure S1: Distribution of farrowing duration, litter size, parity and gestation length.
