# Supplementary figures and images for: Effects of Litter Size and Parity on Farrowing Duration of Landrace × Yorkshire Sows
Source: Animals (Basel). 2021 Dec 31;12(1):94. doi: 10.3390/ani12010094 (PMC8749871; doi:10.3390/ani12010094)

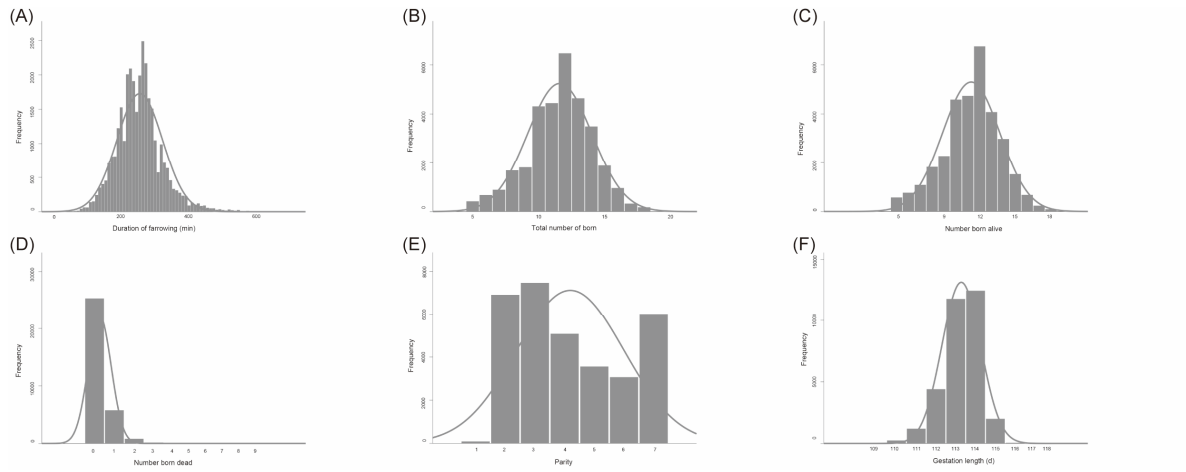

**Figure S1.** Distribution of farrowing duration, litter size, parity and gestation length.

Supplement: Supplementary file 1 [file animals-12-00094-s001.zip › animals-1457212-supplementary.pdf]
